# Adult-Onset Transcriptomic Effects of Developmental Exposure to Benzene in Zebrafish (*Danio rerio*): Evaluating a Volatile Organic Compound of Concern

**DOI:** 10.3390/ijms242216212

**Published:** 2023-11-11

**Authors:** Mackenzie L. Connell, Chia-Chen Wu, Jessica R. Blount, Alex Haimbaugh, Emily K. Kintzele, Dayita Banerjee, Bridget B. Baker, Tracie R. Baker

**Affiliations:** 1 Department of Global and Environmental Health, University of Florida, Gainesville, FL 32610, USA; mconnell@ufl.edu (M.L.C.); emily.kintzele@ufl.edu (E.K.K.); dayitabanerjee@ufl.edu (D.B.); 2 Institute of Environmental Engineering, National Yang Ming Chiao Tung University, Hsinchu City 300093, Taiwan; cchenwu@nycu.edu.tw; 3 Institute of Environmental Health Sciences, Integrative Biosciences Center, Wayne State University, Detroit, MI 48202, USA; jrblount@med.wayne.edu (J.R.B.); alexhaimbaugh@gmail.com (A.H.); 4 Department of Pharmacology, School of Medicine, Wayne State University, Detroit, MI 48201, USA; 5 IFAS Department of Wildlife Ecology and Conservation, University of Florida, Gainesville, FL 32611, USA; bridgetbaker@ufl.edu

**Keywords:** benzene, transcriptomics, volatile organic compounds, zebrafish, adult-onset disease

## Abstract

Urban environments are afflicted by mixtures of anthropogenic volatile organic compounds (VOCs). VOC sources that drive human exposure include vehicle exhaust, industrial emissions, and oil spillage. The highly volatile VOC benzene has been linked to adverse health outcomes. However, few studies have focused on the later-in-life effects of low-level benzene exposure during the susceptible window of early development. Transcriptomic responses during embryogenesis have potential long-term consequences at levels equal to or lower than 1 ppm, therefore justifying the analysis of adult zebrafish that were exposed during early development. Previously, we identified transcriptomic alteration following controlled VOC exposures to 0.1 or 1 ppm benzene during the first five days of embryogenesis using a zebrafish model. In this study, we evaluated the adult-onset transcriptomic responses to this low-level benzene embryogenesis exposure (n = 20/treatment). We identified key genes, including *col1a2* and *evi5b*, that were differentially expressed in adult zebrafish in both concentrations. Some DEGs overlapped at the larval and adult stages, specifically *nfkbiaa, mecr,* and *reep1*. The observed transcriptomic results suggest dose- and sex-dependent changes, with the highest impact of benzene exposure to be on cancer outcomes, endocrine system disorders, reproductive success, neurodevelopment, neurological disease, and associated pathways. Due to molecular pathways being highly conserved between zebrafish and mammals, developmentally exposed adult zebrafish transcriptomics is an important endpoint for providing insight into the long term-effects of VOCs on human health and disease.

## 1. Introduction

Benzene is a prominent volatile organic compound (VOC), which is a group of aromatic or chlorinated organic chemicals commonly found in manufactured products that have high vapor pressure, and thus vaporize readily at room temperature. VOCs are found in the environment, both in the airshed and below ground, predominantly sourced from manufactured products (e.g., building materials, paints/solvents, cleaning agents, furnishings, adhesives, combustion materials, floor and wall coverings) and industries (e.g., petroleum, automotive), including post-industrial cities even after industries become inactive [1,2,3,4]. There are natural sources of VOCs, such as benzene emission from volcanoes and wildfires; however such natural sources of benzene are less prevalent than anthropogenic sources [5]. In the environment, benzene is predominantly found in air, measuring in the range of 0.02 to 34 parts per billion (ppb), with inhalation accounting for 95% of daily human exposure [6,7]. Benzene is also present in contaminated freshwater, groundwater, and other natural water sites [7]. A study of domestic drinking water wells in the United States found benzene concentrations within one order of magnitude below its maximum containment level (5 ppb) and above the goal (0 ppb) set by the Environmental Protection Agency (EPA) [6,8]. Benzene-containing solvents and other materials are commonplace occupational hazards, often found in locations like nail salons, mechanic shops, and manufacturing settings where the burning of plastics is common [9,10]. Benzene exposure pathways also include vehicle exhaust, thermally damaged plastic drinking water pipes, pharmaceuticals, and personal care products [11,12,13,14,15]. For example, benzene is allowable in pharmaceuticals up to 2 parts per million (ppm) [12]. Thus, people are exposed to benzene not only through inhalation, but also ingestion and dermal contact. 

Several VOCs are known carcinogens, linked to prostate cancer, gastrointestinal cancer, leukemia/lymphoma, lung cancer, and bladder cancer, warranting exposure limits imposed by the EPA in the early 1990s [16,17]. The relationship between VOC exposure and cancer outcomes is so strong that, more recently, urinary and exhaled breath VOC biomarkers have been used for the early detection of some cancers [17,18]. Benzene, a group A human carcinogen and established leukemogen, is linked to non-Hodgkin’s lymphoma [16,19,20]. Other acute and chronic health effects linked to benzene specifically include asthma, respiratory diseases, insulin resistance in adulthood, liver and kidney dysfunction, neurologic impairment, and oxidative stress [21,22,23,24]. Additionally, epidemiological studies report linkages between benzene exposure and decreased birth weight, as well as linkages between benzene mixture exposures, specifically benzene, toluene, ethylbenzene, and xylene (BTEX), and preterm birth [25,26]. Maternal–child health outcomes related to VOCs are important endpoints to consider in toxicological studies based on these previous findings and evidence supporting the vulnerability of pregnant women and children to benzene exposure, which is tied to both physiological susceptibility and time spent indoors compared to other groups [21,27,28]. 

Benzene exposure has also been evaluated in mammalian and fish models. Studies in fathead minnows (*Pimephales promelas*) and zebrafish (*Danio rerio*), an established model of developmental toxicity for human translation, suggest that benzene exposure causes physiological abnormalities later in life, hampered embryonic growth, behavioral change, or lethality [29,30]. Benzene is a component of petroleum products and crude oil that are persistent environmental contaminants. Therefore, the findings from studies of diluted bitumen (dilbit) are especially relevant. Results from dilbit toxicity studies using the zebrafish animal model indicated that the toxicity of the petroleum product is majorly driven by VOCs, of which benzene was found to be the most concentrated [31,32]. Another study utilizing zebrafish examined exposure to benzene alone versus BTEX and showed strong evidence that benzene exposure causes significant alterations in phenotypic responses that affect immediate health outcomes at 4 and 10 days post-fertilization (dpf) [33]. Adverse immunotoxicity, hematotoxicity, oxidative stress, and fetal toxicity endpoints have been demonstrated following benzene exposure in mammalian models [34,35,36,37]. Benzene has also been identified as an industrial neurotoxin and can impair neurochemical levels and induce hypothalamic inflammation or insulin resistance known to exacerbate disease development in adulthood [38,39]. Murine models have elucidated the importance of sex-specific effects, and susceptibility to benzene-induced toxicity, particularly in males, as well as differential pathway activation including embryonic signaling pathways [40,41]. 

A common limitation of these previous studies is that VOC exposures, for most people, fall below health-based guidelines, with even occupational exposures in the United States below the Occupational Health and Safety Administration’s (OSHA) permissible exposure limit (PEL) of 1 ppm over 8 h [42]. There are few data published on non-occupational and low-level (<1 ppm) exposures to benzene, with even fewer available on the transcriptomic outcomes of benzene-induced reproductive toxicity [1]. Further, there is a persistent environmental and regulatory concern given that many occupational exposure limits are outdated or not enforced [10]. The purpose of this study was to examine the later-in-life (adult-onset) effects of developmental exposure to low-level concentrations (0.1 and 1 ppm) of benzene. Developmental exposure during embryogenesis (0–5 dpf) is a critical window of development where important body systems are immature. During this window, organisms are especially vulnerable to environmental factors that can change how immune, hematopoietic, reproductive, and other important body systems function, and the development of disease later in life. The zebrafish model is well suited for developmental and reproductive toxicity studies, largely because it is high throughput, cost effective, and because disease-causing genes and pathways are highly conserved with humans, making findings relevant for human health [43]. The immediate phenotypic, transcriptomic, and neurobehavioral effects of developmental benzene exposure have been reported previously [44]. A cohort from these benzene-exposed zebrafish were subsequently raised in the absence of benzene exposure to examine tissue- and sex-specific transcriptomic changes. Investigating the effects observed during adulthood provides the critical next step in determining early life molecular signatures of benzene exposure and potential biomarkers of effect, especially pertaining to reproductive and neurological system functioning. 

## 2. Results

### 2.1. Benzene Exposure

No significant difference in mortality was observed in larvae or adults among the treatments. The exposure water, egg, and larval body burden measured after exposure are published as part of our previous paper [44].

### 2.2. Differential Gene Expression 

**Figure 1:**
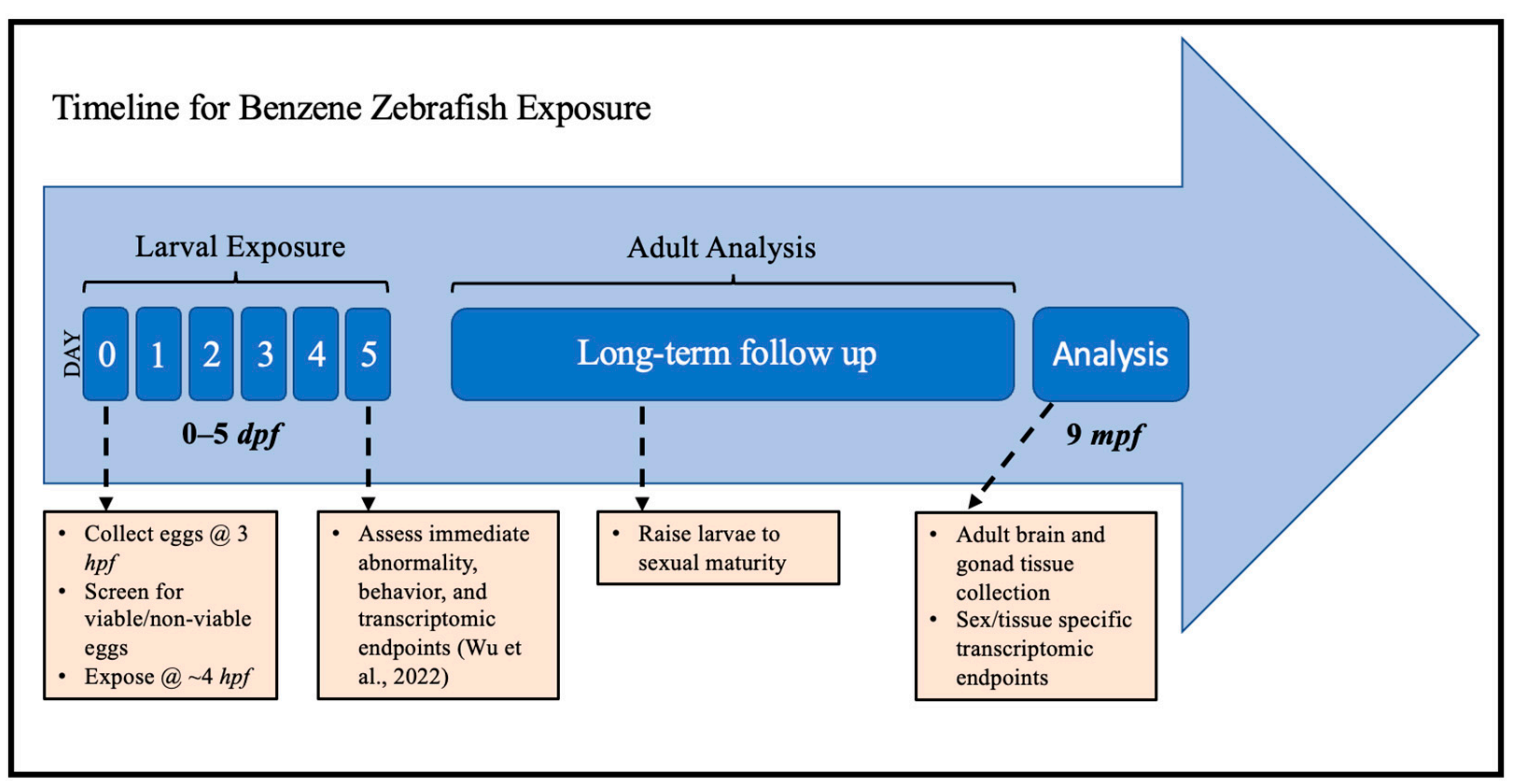
Experimental schema for embryonic zebrafish exposure to benzene and long-term follow-up for transcriptomic analysis in adulthood. hpf: hours post-fertilization; dpf: days post-fertilization; mpf: months post-fertilization. Immediate endpoints were reported in Wu et al., 2022 [44].

**Figure 2:**
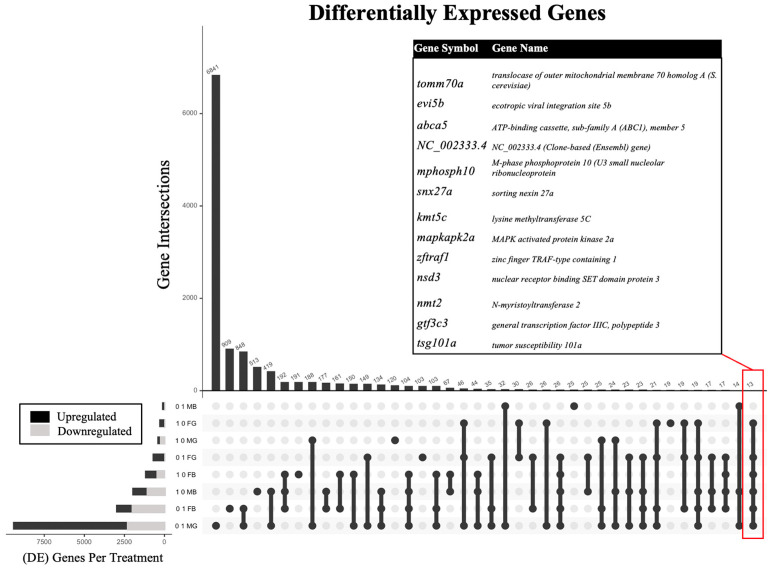
Upset plot [45,46] showing the number of differentially expressed genes (DEGs) and DEG intersections in descending order by frequency for adult zebrafish following embryonic benzene exposure at either 0.1 or 1.0 ppm in male (M) versus female (F) brain (B) or gonad (G). The inset table indicates 13 DEGs common to six of the eight exposure groups.

The sex- and tissue-specific transcriptomic results were obtained according to the study progression timeline (Figure 1) and each benzene concentration was compared to the controls to assess the differential expression of genes. The analysis of the effect of benzene exposure across eight exposure groups compared to control revealed significantly altered gene expression (Table 1). Embryonic benzene exposure at 0.1 ppm resulted in 124 and 2992 differentially expressed genes (DEGs) in adult male and female brains, respectively, as well as 9421 and 713 DEGs in adult male and female gonads, respectively. Embryonic benzene exposure at 1.0 ppm resulted in 1981 and 1187 DEGs in adult male and female brains, respectively, as well as 415 and 296 DEGs in adult male and female gonads, respectively. A total of 13 DEGs were common to six of the eight exposure groups (Figure 2). This included the *abca5*, *gtf3c3*, *nmt2*, and *mapkapk2a* genes. For *abca5*, dysregulation was observed in 0.1 ppm female brain (*p* = 0.002), 0.1 ppm female gonad (*p* = 0.008), 1.0 ppm female brain (*p* = 0.0001), 1.0 ppm female gonad (*p* = 0.01), 1.0 male brain (*p* = 0.01), and 0.1 ppm male gonad (*p* > 0.05). The gene *gtf3c3* was dysregulated in 0.1 ppm female brain (*p* = 0.03), 0.1 ppm female gonad (*p* = 0.001), 1.0 ppm female brain (*p* = 0.02), 1.0 ppm female gonad (*p* = 0.009), 1.0 ppm male brain (*p* = 0.009), and 0.1 ppm male gonad (*p* > 0.05). The gene *nmt2* was most altered in all female groups and the 1.0 ppm male brain (*p* < 0.05, fc > 0.5). Lastly, *mapkap2ka* was perturbed in the 0.1 ppm female brain, 0.1 ppm female gonad, 1.0 ppm female brain, 1.0 female gonad, and 1.0 ppm male brain (*p* < 0.05, fc > 0.5).

One gene was differentially expressed across all four male exposure groups: collagen type-I alpha 2 (*col1a2*), with a log2 fold change for the gonad of 1.29 (*p* = 0.003) and 0.61 (*p* = 0.024), following 0.1 and 1 ppm exposure, respectively; and –0.57 (*p* = 0.015) and 0.91 (*p* = 0.016) for brain, following 0.1 and 1 ppm exposure, respectively (Table 2). Forty DEGs were common across all four female exposure groups.

**Figure 3:**
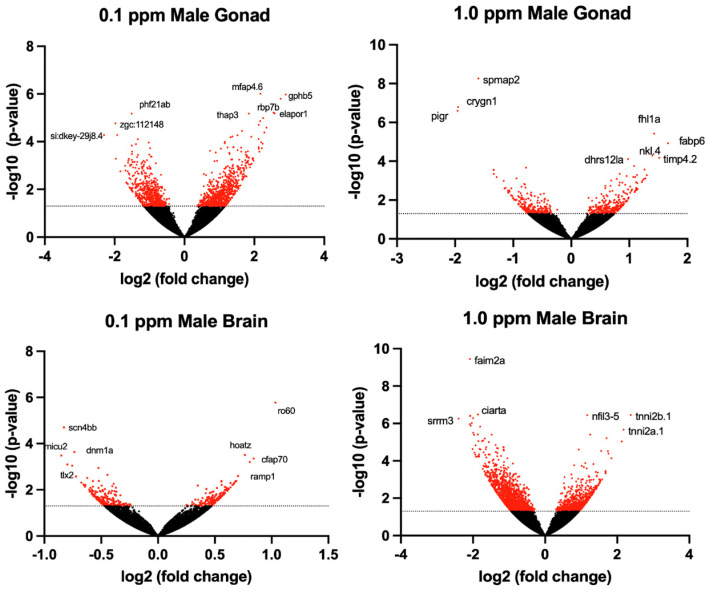
Volcano plots illustrate the differentially expressed genes (DEGs) in adult male zebrafish following embryonic benzene exposure. DEGs with *p* < 0.05 denoted in red. The dashed line indicates the value 1.3.

**Figure 4:**
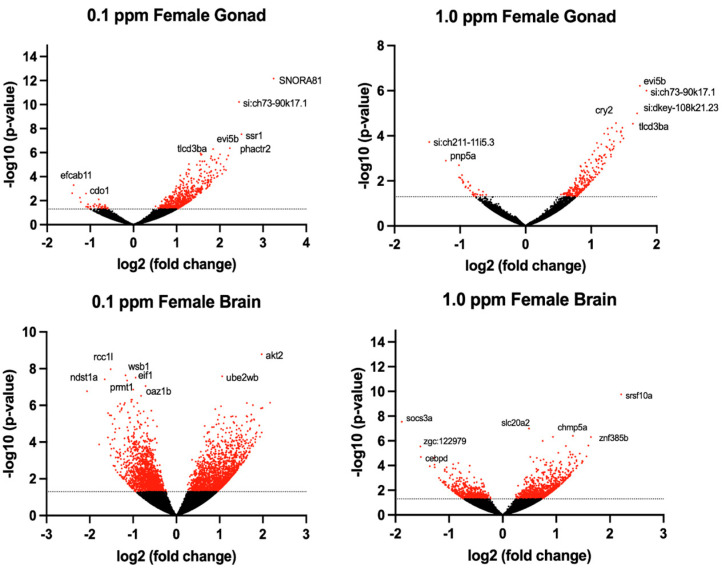
Volcano plots illustrate the differentially expressed genes (DEGs) in adult female zebrafish following embryonic benzene exposure. DEGs with *p* < 0.05 denoted in red. The dashed line indicates the value 1.3.

The transcriptomic profile of female ovarian tissue was significantly altered by benzene exposure, with 490 DEGs unique to 0.1 ppm benzene exposure, 73 DEGs unique to 1.0 ppm benzene exposure, and 223 DEGs common to both exposure groups (*p* < 0.05). Similarly, in testes, there were 9155 DEGs unique to 0.1 ppm benzene exposure, 149 DEGs unique to 1.0 ppm benzene exposure, and 266 DEGs common to both exposure groups (*p* < 0.05). For both male and female gonads, differential gene expression was skewed towards overexpression (Figure 3 and Figure 4). The DEGs in female gonad were 95% and 89% upregulated for 0.1 and 1.0 ppm benzene, respectively. This skew was evident, but slightly less pronounced in male gonad, being 76% and 55% upregulated for 0.1 ppm and 1.0 ppm.

Several significant DEGs are related to the top altered pathways identified using pathway analysis (the results of which are reported below). The TLC domain containing 3Ba (*tlcd3ba*) was upregulated in the female brain at 0.1 (fold change (fc) = 0.529, *p* = 0.025) and 1.0 ppm (fc = 0.658, *p* = 0.001) benzene, but it was more significantly upregulated in the female gonad, at both 0.1 (fc = 1.553, *p* = 0.000006) and 1.0 ppm (fc = 1.379, *p* = 0.0002) benzene. All gonad tissues showed the significant dysregulation of cdc42 effector protein (Rho GTPase binding) 5 (*cdc42ep5*), hypermethylated in cancer 1-like (*hic1l*), and chemokine (C-X-C motif), receptor 4b (*cxcr4b*) (fc > 0.5, *p* < 0.05). The genes associated with benzene exposure in the female gonad are ecotropic viral integration site 5b (*evi5b*), *tlcd3ba*, and *si:ch73-90k17.1* (Table 2). We also identified three genes in signaling pathways that were linked to the *mitogen-activated protein kinase* (MAPK) cascade: MAPK-activated protein kinase 2a (*mapkapk2a*), B-Raf proto-oncogene, serine/threonine kinase (*braf*), and pre-mRNA processing factor 4Ba (*prpf4ba*) (Table 2). Genes that were also relevant to previous larval studies [44] include receptor accessory protein 1 (*reep1*), mitochondrial trans-2-enoyl-CoA reductase (mecr), nuclear factor of kappa light polypeptide gene enhancer in B-cells inhibitor, alpha a (*nfkbiaa*), TAF4A RNA polymerase II, TATA box binding protein (TBP)-associated factor (*taf4a*), glutamate receptor, ionotropic, kainite 1a (*grik1a*), assembly factor for spindle microtubules (*aspm*), and matrix Gla protein (*mgp*) (Supplemental Table S1).

### 2.3. Pathway Analysis

Pathway analysis uncovered the top altered pathways associated with developmental benzene exposure in adult zebrafish brain and gonad tissue. The pathways were involved in diseases and disorders, notably cancer, which was evident in every exposure group following 0.1 or 1.0 ppm embryonic benzene exposure (Supplemental Table S2). 

Specifically following 0.1 ppm benzene exposure, the top altered pathways in male brain were cancer, followed by neurological disease, organismal injury and abnormality, reproductive system disease, and endocrine system disorders (Supplemental Table S2). The top altered pathways for female brain also included cancer, followed by organismal injury and abnormality, endocrine system disorders, gastrointestinal disease, and neurological disease, in that order. Gonad tissue for males and females revealed the same top five diseases and disorders: cancer, organismal injury and abnormality, endocrine system disorders, gastrointestinal disease, and neurological disease. 

Following 1.0 ppm benzene exposure, the top disorders identified through pathway analysis for both male brain and gonad were cancer, organismal injury and abnormality, endocrine system disorders, and reproductive system disease (Supplemental Table S2). The top diseases and disorders for female brain were cancer, gastrointestinal disease, organismal injury and abnormalities, endocrine system disorders, and hereditary disorders; for female gonad, they were cancer, organismal injury and abnormality, gastrointestinal disease, endocrine system disorders, and reproductive system disease (Supplemental Table S2). 

**Figure 5:**
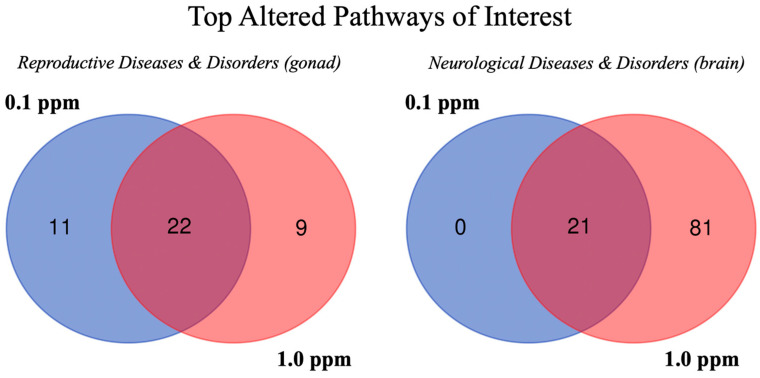
Venn diagrams showing the number of altered pathways related to reproductive system or neurological diseases and biofunctions in adult zebrafish gonad or brain following developmental benzene exposure.

Pathway analysis revealed 28 different reproductive system diseases and biofunctions as altered based on observed molecular expression and/or phosphorylation changes, 15 of which were impacted in both brain and gonad for both sexes at both benzene exposure concentrations: endometrial adenocarcinoma, breast or ovarian carcinoma, female genital carcinoma, breast or gynecological cancer, development of genital tumor, endometrial cancer, uterine tumor, female genital tract cancer, female genital neoplasm, uterine cancer, female genital tract adenocarcinoma, uterine carcinoma, breast cancer, ovarian cancer, and tumorigenesis of the reproductive tract. The development of a genital tumor (molecule number range = 63–1068) and tumorigenesis of the reproductive tract (molecule number range = 63–1088) were analyzed at a molecular level (Table 2) due to the total number of functional molecules present and predictive values associated with them. The absolute top activated or inhibited reproductive pathways of interest and corresponding activation z-scores from the gonad tissue results are reported in Table 3 and Supplemental Table S3. When just the gonad tissue was considered, there were 22 reproductive system disease pathways altered in male and female groups at both concentrations (Figure 5).

Pathways associated with neurological diseases and disorders were altered in the brain tissue. In brain tissue specifically, there were 21 altered sub-pathways in male and female groups at both concentrations that were associated with neurological development or behavior disorders (Figure 5). One neurological sub-pathway of interest, nervous system neoplasm, was altered in male and female brain tissue at both concentrations, but these were not altered enough to predict activation or inhibition (z-score < |2|). Although there were neural effects indicated at the pathway level, these were less abundant and significant than the reproductive effects concluded based on pathway analysis. Reproductive and neurological pathways from gonad or brain tissues (activation z-scores) are reported in Table 3.

The NRF2-mediated oxidative stress response pathway was altered in female brain following 0.1 (−log(*p*-value) = 2.88, z-score = 1.46) and 1.0 ppm (−log(*p*-value) = 3.05, z-score = −0.28) benzene exposure, and female gonad following 0.1 ppm (−log(*p*-value) = 0.365, z-score = 2) benzene exposure. The insulin secretion pathway was activated most significantly for female gonadal tissue following exposure to both concentrations: 0.1 ppm (z-score = 3.207) and 1.0 ppm (z-score = 2.23). Further pathway analysis of the gonad tissue identified three endocrine system functional pathways that were perturbed in all groups (Supplemental Table S3).

## 3. Discussion 

To our knowledge, this is the first study to investigate tissue- and sex-specific transcriptomic outcomes in adulthood following low-level, embryonic benzene exposure. Using a zebrafish model, we determined that the top altered pathways in adult zebrafish brain and gonad following embryonic benzene exposure, regardless of sex, included cancer, reproductive system disease (notably, the development of genital tumor and tumorigenesis of the reproductive tract), and neurological disease. Another novel and important observation was sex-dependent effects, and for most conditions, the lower (0.1 ppm) exposure dose had significantly more differentially expressed genes than the 1.0 ppm dose.

Benzene is a known carcinogen that has also been linked to reproductive health outcomes [26]. It is not surprising that pathway analysis revealed that most of the reproductive system disease pathways were associated with cancer. Incidence of breast cancer and endometrial cancer/adenocarcinoma were two of the most affected pathways. Breast cancer has been linked to benzene/VOC exposures in humans and rodent models, but endometrial cancer has yet to be studied as an endpoint in benzene exposure studies in humans or rodents. Endometrial cancer risk has been linked to tobacco smoking, a common source of VOC exposure, but, converse to our findings, some studies have attributed a protective association [47]. In human studies of breast cancer, the literature is mostly focused on occupational exposure to benzene and generally points to benzene exposure as a risk factor for breast cancer [48,49,50]. Looking specifically at the gonad tissue, we found that genital tumor and reproductive tract tumor pathways were more important pathways of note that relate to the previous toxicological assessment of benzene [51]. It is important to note that few studies focus on low-level exposure, which is likely linked to sub-cancerous outcomes. Reproductive pathways independent of cancer were observed, especially in low-concentration benzene gonad tissue. Pathways linked to the morphology of genital organs and atrophy of the testis were observed at 0.1 ppm benzene exposure, but were not found in higher (1.0 ppm) exposure groups. This could indicate effects on reproductive functioning yet to be observed in other studies that are focused on higher benzene concentration exposures and cancerous outcomes. This emphasizes a gap in the literature for the study of non-cancer outcomes, such as chronic diseases related to benzene exposure, which one review has previously identified [52].

The evaluation of neurological pathways specifically associated with brain tissue revealed that central nervous system function, learning and memory, and neuron development were the most highly affected across groups. Nervous system neoplasm was significantly affected for all brain tissues, with scores nearing an increased activation of the pathway. Nervous system neoplasm has been linked to VOC exposure in human studies, but the relationship is not well defined. One link has been established in a cohort of children exposed to ambient residential or traffic-related air pollution, including benzene [53,54]. Central nervous system cancer and disease pathways were also consistently affected. In the 1.0 ppm concentration groups, learning, memory, cognition, and neuron development were highly affected. This finding is consistent with related rodent models and humans where there is evidence for prenatal exposure to benzene and impaired cognition of offspring [55,56,57]. Many of the pathways altered by lower-level benzene exposure were also altered at the higher concentration and very few pathways related to disease were altered in the lower concentration groups (male or female) alone.

The endocrine system pathways were affected in the gonad tissue of both sexes and concentrations, though to a lesser extent than the reproductive and neurological pathways. The endocrine pathways that were altered most frequently were thyroid carcinoma, nonpituitary endocrine tumor, and endocrine carcinoma. Benzene has gained traction and a growing body of evidence suggesting its endocrine-disrupting potential. Recently, it has been proposed that many VOCs (including benzene) have possible endocrine-disrupting effects at levels lower than would induce carcinogenicity. Many endocrine system disorders involve thyroid development and pancreatic function. Surprisingly, after pathway analysis comparing the 0.1 and 1.0 ppm benzene concentrations, there were no activated or inactivated canonical pathways that overlapped between the two condition groups.

Thirteen DEGs were altered across multiple benzene exposure concentrations, regardless of sex or tissue. Notably, *abca5* and *gtf3c3* were significantly altered in female brain at both exposure concentrations and in male brain at 1.0 ppm. *ABCA5*, the human ortholog, has functionality for peptide production with potential effects for late-onset Alzheimer’s disease in humans and mice [58]. Recently, *ABCA5* has also been called upon as a potential new biomarker of colorectal cancer, a health outcome that has also been associated with benzene exposure [59,60]. While the role of *gtf3c3* is largely undetermined, several studies support its candidacy for neurodevelopmental disorders, namely, epileptic encephalopathy and intellectual disability [61,62]. Also notably altered in many groups were *nmt2* and *mapkapk2*. *NMT2/Nmt2* is relatively understudied in human and murine genetics, but is expressed in the testis and during embryonic development, with case studies pointing to a link between the disruption of *NMT2*/*Nmt2* and hypoplastic testes [63,64]. The disruption of *nmt2* resulting from benzene exposure should be evaluated further as it relates to the underdevelopment of the gonad. *Mapkapk2a*, orthologous to human *MAPKAP2*, plays a role in spermatogenesis in mice and is essential for development [65,66]. Interestingly, these genes have more profound implications for male reproductive development than for females, where the data from the current study show more of an effect on female tissues at both exposure concentrations. The significant dysregulation of *abca5*, *gtf3c3, nmt2,* and *mapkapk2a* in the current study justifies future studies investigating the link between environmentally relevant exposures to benzene during critical windows and the neurodevelopmental or reproductive effects present in adulthood and in subsequent generations. 

Some of the most significant DEGs were found in both male and female gonad, notably *cdc42ep5* and *cxcr4b*. *Cdc42ep5* is a septin modulator that is important for the motility of cancer cells and has been identified in networks (CDC42EP5-SEPT9 axis) that determine the aggressiveness of some cancers like melanoma [67]. Similarly, the overexpression of *CXCR4* has been associated with cancer (breast, colorectal) and exacerbated cancer outcomes (metastasis, tumor growth, relapse), and has been studied as a target for cancer therapies [33,68]. Evidence for the link between the overexpression of *CXCR4* and *CDC42EP5* and cancer has been established in the literature, but the link between benzene exposure and diminished reproductive capacity is still debated [33,69]. Data from Diotel et al. (2010) also provide evidence for the role of *cxcr4b* in the neural development of zebrafish, building on previous work that established the expression of *cxcr4b* in the zebrafish midbrain [70,71]. However, in the current study, the significant upregulation of cxcr4b was only observed for female brain following 0.1 ppm benzene exposure, and thus could play a role in neurobehavioral outcomes of benzene exposure at the concentrations studied here.

In female gonad, three genes were significantly dysregulated by both 0.1 and 1.0 ppm benzene: *evi5b*, *tlcd3ba*, and *si:ch73-90k17.1*. Overall, the differential gene expression in exposed females was skewed towards upregulation in the gonad. *Evi5b* is orthologous to the human gene *EVI5*, a noted potential oncogene and cell cycle regulator [72]. Evidence suggests that *EVI5* is an important risk gene for diseases such as multiple sclerosis and leukemia in humans and murine models [73,74]. Given the risk associated with overexpression, *EVI5* and family members are important prognostic markers for survival and cancer recurrence [75]. The perturbation of *evi5b* in all female groups highlights the potential role of this gene in cancer and reproductive system disease outcomes. *Tlcd3ba* was significantly upregulated in female gonad, particularly following 0.1 ppm benzene exposure. This is a novel finding with no previous evidence suggesting the role of *tlcd3ba* in the reproductive tract. Not surprisingly, *tlcd3ba* (previously *fam57ba*) was also upregulated in female brain, which is known to be expressed in the brain and nervous system of zebrafish and is associated with retinal dystrophy in mice and humans [76,77]. The last gene significantly dysregulated, *si:ch73-90k17.1*, is understudied and is therefore unable to be contextualized here given the minimal information available.

The most DEGs across all exposed groups were found in male gonad following 0.1 ppm benzene exposure, with *col1a2,* a gene within the development of a genital tumor and tumorigenesis of the reproductive tract pathways, being differentially expressed across all male exposure groups. The human ortholog, *COL1A*, is implicated in inherited connective tissue disorders, including osteogenesis imperfecta (OI) (multiple), perinatal lethal OI, and Ehlers–Danlos syndrome (EDS) that can be passed to offspring [78,79]. Data from murine models with *Col1a2* mutations provide further evidence that OI impairs prenatal uterine function in a significant number of affected fetuses [80]. In humans, EDS also has profound reproductive effects, specifically when the offspring is affected, but the mother is not affected by the connective tissue disorder [81,82]. The role of EDS in reproductive outcomes is not well understood, but population-based studies have reported an elevated number of premature rupture of membranes in the fetus and subsequent preterm birth outcomes [81,83,84]. *COL1A2* also has neural impacts and is implicated in intercranial aneurysm, and, in fact, there was elevated expression in male brain at both benzene exposure concentrations [85]. Further research is required to uncover the mechanistic plausibility of benzene-induced alterations to *col1a2* and subsequent reproductive effects, particularly in males. 

Conversely, across female exposure groups, there were 21 DEGs associated with the development of a genital tumor and tumorigenesis of the reproductive tract pathways, including *evi5b* and genes in the MAPK cascade (i.e., *mapkapk2, braf,* and *prpf4b*). The biological functions of the MAPK cascade include critical roles in initiating cell development, proliferation, and differentiation, with the overactivation of this pathway being the cause of many human cancers [86,87]. The activation of the MAPK pathway occurs as a result of transmitted stimuli that include, but are not limited to, metabolic stress, DNA damage, or growth factors [87]. *Mapkapk2* is involved in cellular processes ranging from inflammatory responses to roles in tumorigenesis and spermatogenesis [65]. *Braf* is known to precede MAPK in the signal transduction pathway, and mutations of this gene (BRAF^V600E^) are found in several cancers (e.g., thyroid, melanoma, and leukemia) and can activate the MAPK pathway independent of other stimulation. *Prpf4b* has homology to MAPKs, but is more commonly known as a splicing factor that is necessary for cancer cell migration and its role in human primary breast tumors [88].

The findings here also show that the insulin secretion pathway was activated for gonadal tissue of both concentrations, although this finding was more evident in female groups and in the brain tissue specifically. This has important implications for offspring, as a similar murine study showed that maternal benzene exposure impacted insulin resistance for the next generation, particularly predisposing male offspring [89]. Another study of the reproductive toxicity of benzene noted that the metabolic imbalance and hyperinsulinemia associated with exposure to benzene in adulthood was treatable with an anti-diabetes drug [90]. Both studies only conducted inhalation exposure during adulthood rather than developmentally, which could account for the differences with the current study. These studies, particularly regarding the offspring of benzene-exposed mice, open the door for transgenerational zebrafish studies and the metabolic effects associated with benzene.

These results point to sex-dependent effects associated with benzene exposure. When considering the overall number of DEGs, males were more affected than females at the higher, 1.0 ppm, concentration of benzene with less pronounced sex differences at the lower (0.1 ppm) concentration. All tissues had more DEGs in males versus females, except for the lower level (0.1 ppm) brain, which had far more DEGs in the females. Previous research shows that male animals are more highly afflicted by benzene exposure with respect to genotoxicity and hematotoxicity, which could explain the increase in the gene expression changes described here [91]. The evaluation of DEGs also elucidated that in most tissue, the very low dose (0.1ppm) was significantly more affected, with a higher number of DEGs, than the 1.0 ppm exposure. This an important finding and is likely due to the fact that we are looking at adult-onset (around 9 months later) changes in gene expression. For the larval acute doses {44}, we did see a dose-dependent effect, but the effects these chemicals cause later in life are not so straightforward. As we continue to find non-monotonic responses to multiple contaminants at low levels, and later in life, there is a real need to focus more closely on these changes. 

The immediate transcriptomic response and pathway analysis in larval zebrafish to embryonic benzene exposure at the same two concentrations investigated in this study have been reported by our lab previously [44], in which we found similarities between the larval and adult life stages. Cancer is the top altered pathway across the life stages, followed by organismal injury and abnormalities, organismal survival, and nervous system development and function (Supplemental Table S2) [44]. Differential activation of the insulin secretion signaling pathway was also conserved across the life stages.

Altered expression in both (0.1 ppm and 1.0 ppm) larval groups as well as both concentrations in adult female gonad tissue and 1.0 adult male brain was found for the *reep1* gene. Upregulation occurred in all but the 1.0 ppm male brain group, which was downregulated. Mitochondrial dysfunction, reduced ATP production, increased susceptibility to oxidative stress, and impaired locomotor activity (zebrafish) have all been linked to *reep1* [92]. The linkage to reactive oxidative stress is congruent with previous findings for benzene concentrations of 1 ppm and above, but there is less evidence that this link exists at levels below 1.0 ppm benzene. Further, the findings from this investigation are consistent with previous findings linking benzene exposure and an induced oxidative stress response [93]. Specifically, for benzene-exposed females, the alteration of the NRF2-mediated oxidative stress response pathway was observed. Recent findings have linked benzene exposure to the activation of the NRF2 signaling pathway with downstream implications for ferroptosis (regulated by the oxidative stress pathway) in a murine hematotoxicity model [40]. Additionally, in previous research, nonacute toxic benzene and toluene levels were shown to enrich the proteins involved in NRF2-mediated oxidative stress of lung epithelial cells (A549), indicating a strong link between benzene exposure and oxidative stress responses [94]. This study provides evidence for this low-level oxidative stress response to some extent, especially for 0.1 ppm female tissues. Surprisingly, significant locomotor changes were not reported in the previous study’s larval population, but were reported in the adult 1.0 ppm male and female groups (brain). This further justifies a need to assess this endpoint in adulthood, as it could develop later in life. 

Another commonly altered gene in the benzene-exposed larval and adult populations was *mecr*. The mitochondrial trans-2-enoyl-CoA reductase (*mecr*) gene was significantly altered and has reproductive and cardiac implications. Evidence from a knockout mouse model has proposed that knockout-first-type mutations to *Mecr* display embryonic lethality and pantropic effects on placental development due to deficient mitochondrial respiration because of disrupted mitochondrial fatty acid synthesis [95]. In contrast, murine models with overexpressed *Mecr* developed cardiac abnormalities and hereditary cardiomyopathy [96].

*Nfkbiaa* was more perturbed in the adults of the low-level developmental exposure to benzene compared to larval exposure and was altered in female 0.1 ppm brain and gonad tissues as well as 0.1 male gonad and 1.0 male brain. Conversely, *nfkbiaa* was upregulated in the higher (1.0 ppm) concentration larval transcriptomic results [44]. Involved in overall cell survival, *nfkbiaa* is a key indicator of development. Further, recent results point to *nfkbiaa* as a target of miR-202-3p, a microRNA associated with embryo development at the mid-blastula stage [97]. These findings suggest that the over- or underexpression of *nfkbiaa* can cause developmental delay in zebrafish. The consistency of this finding in immediate and long-term gene expression may suggest later-in-life developmental and reproductive implications tied to benzene exposure. Other key genes with altered expression in the larval population and adult population were *taf4a*, *grik1a*, *aspm*, and *mgp* (Supplemental Table S1). 

One limitation of the current study was the inability to assess fertility and adult-onset behavioral responses that may be associated with benzene exposure. With a larger original cohort, a subset of adult zebrafish could be allocated for these assays. Similarly, the histology of reproductive organs could allow the observation of tumor or hypoplasia, as indicated by the results here. Future studies are needed to determine whether these adult-onset transcriptomic responses have long-term consequences epigenetically or phenotypically at levels equal to or lower than 1 ppm. 

## 4. Materials and Methods

### 4.1. Animal Husbandry 

Adult AB strain zebrafish were housed in buffered reverse osmosis (RO) water (60 mg/L Instant Ocean Salts; Aquarium Systems, Mentor, OH, USA) at 28 °C on a recirculating system at a maximum density of 5 fish per liter with a standard light/dark cycle (14/10 h), as previously described [44]. The zebrafish were fed a mixture of Zeigler Adult Zebrafish Diet (Zeigler Bros. Inc., Gardners, PA, USA), Spirulina Flake Fish Food (Ocean Star International, Snowville, UT, USA), 300–500-micron Golden Pearls (Aquatic Foods Inc., Fresno, CA, USA), and supplemental brine shrimp (Artemia International, Fairview, TX, USA) twice daily. The animal use protocols were approved by the Institutional Animal Care and Use Committee at Wayne State University according to the National Institutes of Health Guide to the Care and Use of Laboratory Animals (Protocol No. #IACUC-19-02-0938). 

### 4.2. Spawning 

To obtain zebrafish embryos, zebrafish spawns were initiated the night prior to benzene exposure at a ratio of 2 females per 1 male with plastic dividers between the sexes. The dividers were removed the following morning, and embryos were collected within 4 h. The embryos were rinsed thoroughly with RO water and incubated in egg water (RO water with 0.6 g/L Instant Ocean Salts) containing 0.6% bleach for 10 min. The embryos were then rinsed with RO water again to remove the bleach solution and moved to Petri dishes containing egg water. The embryos were then separated into 30 mL glass vials with a density of 20 embryos per vial containing 20 mL egg media. The exposure included 5 vials per concentration (n = 100 embryos per concentration). 

### 4.3. Benzene Exposures 

Solutions of 0.1 ppm and 1 ppm were made from a stock solution of 99.8% benzene (99.8%, Sigma Aldrich, St. Louis, MO, USA). The 99.8% benzene stock (Sigma Aldrich, St. Louis, MO, USA) was serially diluted in a septum-sealed amber vial with 40 mL egg water to create 998 ppm and 99.8 ppm benzene stock dilutions. In preparation for benzene exposure, 20 uL of egg water was removed from the vials housing embryos to balance the 20 uL of stock dilutions that were injected into vials with a gastight syringe for final concentrations of 0.1 or 1 ppm. To minimize benzene loss through volatilization, the benzene stock dilutions were mixed fresh daily, and the caps were never removed from the septum-sealed vials during or after benzene injection into vials housing embryos (i.e., exposure vials). The control vials contained only 20 mL egg water. The control and exposure vials were then gently rocked by hand and placed in a 28 °C incubator. Over the following 4 days, this procedure was repeated daily to refresh the stock dilution or egg water (Figure 5). Each day, dead embryos/larvae were removed. At 5 dpf, the larval fish were rinsed with egg water 3 times to end the exposure at the end of embryonic development. The larval fish were then moved to six-well plates at 28 °C and fed daily with a daily water change until 7 dpf, at which time the larval fish were relocated to a recirculating system to be raised past sexual maturity (>4 months post-fertilization) under standard husbandry conditions (Figure 5). Replicates were carried out in the larval exposures consisting of 3 separate biological replicates (exposures were conducted identically at 3 different times) and multiple technical replicates within these groups. These 3 replicates (published as Wu et al., 2022) [44] showed no statistical difference in evaluated outcomes, and only one of these replicates was carried forward to the adult stage for further analysis. Body burden measurements of benzene in embryos and solution were performed at Ann Arbor Technical Services (Ann Arbor, MI, USA) and reported in our previous study [44].

### 4.4. Tissue Collection 

After sexual maturity and at the point of adulthood (9 months post-fertilization), adult fish were euthanized in a solution of 0.4 g/L tricaine methane sulfonate (MS-222) buffered with 0.66 g/L sodium bicarbonate. For each exposure concentration and control group, 5 adult female and 5 adult male fish were euthanized and dissected to obtain brain and gonad, resulting in a total of 6 conditions: control males, control females, 0.1 ppm males, 0.1 ppm females, 1.0 ppm males, and 1.0 ppm females. When tissue was considered (2 tissues per animal), the 6 conditions resulted in 12 groups for downstream analysis. Dissections were performed under dissecting microscopes with disinfected instruments on sterile plates. The tissues were immediately deposited into RNAlater (Invitrogen, Carlsbad, CA, USA) for preservation. 

### 4.5. RNA Collection and Isolation 

Following tissue collection, 300 μL RNAlater (Invitrogen, Carlsbad, CA, USA) was added, and the samples were stored at −80 °C until RNA isolation. The samples were removed from −80 °C and allowed to thaw briefly on ice. Upon thawing, the RNAlater was removed and the samples immediately proceeded to the RNA isolation stage. For RNA isolation, the RNeasy^®^ Lipid Tissue Mini Kit (Qiagen, Hilden, Germany) was used according to the manufacturer’s instructions. A Qubit^®^ 2.0 Fluorometer and Qubit^®^ RNA High Sensitivity Assay Kit (Invitrogen, Carlsbad, CA, USA) were used to determine the RNA concentration. The RNA samples were kept at −80 °C until QuantSeq library preparation. 

### 4.6. RNA-Seq QuantSeq

Next, 3′ mRNA-seq libraries were prepared from isolated RNA using the QuantSeq 3′ mRNA-Seq Library Prep Kit FWD for Illumina (Lexogen, Vienna, Austria), according to the manufacturer’s instructions. The samples were amplified at 17 cycles. The libraries were quantified using a Qubit**^®^** 3.0 Fluorometer and Qubit**^®^** dsDNA Broad Range Assay Kit (Invitrogen, Carlsbad, CA, USA), and run on an Agilent TapeStation 2200 (Agilent Technologies, Santa Clara, CA, USA) for quality control. The samples were sequenced on a HiSeq 2500 (Illumina, San Diego, CA, USA) in rapid mode (single-end 75 bp reads). The sample size was n = 5 for all except two groups: n = 4 for female gonads and female brains collected from fish exposed to 1.0 ppm benzene, because one sample from each of these groups was unidentifiable and, therefore, excluded from analysis after sequencing. Reads were aligned to *D. rerio* (Build danRer11) using the BlueBee Genomics Platform (BlueBee, Rijswijk, The Netherlands). The differential gene expression between the control and exposure zebrafish was evaluated using DEseq2 (available through GenePattern; Broad Institute, Cambridge, MA, USA). Genes with significant changes in expression, as defined by an absolute log2 fold change value ≥ 0.5 and *p*-value < 0.05, were uploaded into Ingenuity Pathway Analysis software version 22.0.2 (IPA; QIAGEN Bioinformatics, Redwood City, CA, USA) for analysis using RefSeq IDs as identifiers. The complete list of significant gene data is included in the Supplemental Information. The significant canonical pathways and associated diseases and biofunctions were defined as having *p*-values < 0.05, (or −log10(*p*-value) > 1.3) based on the right-tailed Fisher’s exact test. The z-score was calculated through IPA functionality to predict the activation (≥2) or inhibition (≤−2) of a pathway. 

## 5. Conclusions

Overall, the benzene-induced transcriptomic results shown here indicate profound effects on genes and pathways that regulate cancer, cancer outcomes, neurodevelopment, and reproductive capacity in a sex- and concentration-dependent manner. Gene mutations with strong linkages to downstream fertility and birth outcomes, especially *col1a2*, were especially relevant for the benzene-exposed males. Female gonad gene expression was skewed towards overexpression. Reproductive-disease-associated pathways, including the development of a genital tumor and tumorigenesis of the reproductive tract, were significantly altered across all tissues and benzene concentrations. The genes implicated in delayed neurodevelopment, including *nfkbiaa*, and *reep1*, showed a high level of change post-exposure to benzene, a result that was consistent at both the larval and adult stage. Thus, neurological outcomes were impacted both immediately and later in life. This study identifies areas for future research directions including the investigation of phenotypic, behavioral, and fertility endpoints in adulthood as well as transgenerational effects related to low-level benzene exposure. Furthermore, the evidence provided here shows that very-low-level benzene exposure induces gene expression changes that have cancer, neurodevelopmental, and reproductive associations later in life. 

## Figures and Tables

**Table 1:** Number (#) of differentially expressed genes (DEGs) in adult zebrafish following embryonic benzene exposure using a log2 fold change cutoff of ±0.5 and *p* ≤ 0.05.

Exposure Concentration (ppm)	Sex	Tissue	# DEGs
0.1	Female	Brain	2992
0.1	Male	Brain	124
1.0	Female	Brain	1187
1.0	Male	Brain	1981
0.1	Female	Gonad	713
0.1	Male	Gonad	9421
1.0	Female	Gonad	296
1.0	Male	Gonad	415

**Table 2:** Genes implicated in pathways associated with the development of genital tumors or tumorigenesis of the reproductive tract in adult zebrafish following developmental benzene exposure in male (M) versus female (F) brain (B) or gonad (G). Fold change reported for genes with *p* < 0.05.

Gene Symbol	0.1 ppm				1.0 ppm			
	FB	MB	FG	MG	FB	MB	FG	MG
*col1a2*		−0.57		1.29		0.91		0.61
*mapkapk*	0.66		1.22		0.52	−0.51	0.84	
*larp7*	0.78		1.34		0.59		1.23	
*gtf3c3*	0.53		1.39		0.58		1.28	
*evi5b*	0.87		1.85	0.84	0.88	−0.82	1.74	
*prpf4bb*	0.76		0.87		0.57	−0.59	0.74	
*cyhr1*	0.64		1.37		0.62	−0.76	1.01	
*tlk1a*	0.98		1.43		0.69		0.80	
*zbtb16a*	0.93		0.71		0.76		1.08	
*rev31*	1.03		1.35		0.76	−0.68	0.79	
*garre1*	0.90		1.56		0.94	−0.76	0.79	
*nsd3*	0.83		1.66		0.75	−0.90	0.90	
*nmt2*	0.71		0.95		0.62	−0.96	0.90	
*phrf1*	0.89		1.40		0.59	−0.63	1.08	
*snx27*	1.32		1.66		1.08	−0.88	1.06	
*abca5*	0.86		0.89		0.94	−0.83	0.80	
*ankrd12*	0.74		1.50		0.58		1.05	
*rsf1b.1*	0.80		1.36		0.62	−0.56	1.21	

**Table 3:** Sub-pathways associated with reproductive system, neurological, and endocrine system diseases and disorders altered in gonad or brain tissue after developmental exposure to 0.1 ppm or 1.0 ppm benzene. Activation z-score indicated numerically and *p*-value < 0.05 indicated by * with ns denoting ‘not significant’ for the given condition. Full table can be found in Supplemental Table S3.

Pathway	0.1 Female	1.0 Female	0.1 Male	1.0 Male
*Reproductive Diseases and Disorders (Gonad)*				
Genital tumor	−0.439 *	1.154 *	1.079 *	2.59 *
Pelvic tumor	−0.595 *	1.154 *	0.819 *	2.581 *
Endometrial cancer	*	*	*	*
Female genital tract cancer	*	*	*	*
Breast or ovarian carcinoma	−1.067 *	*	1.066 *	*
Breast or gynecological cancer	−1.387 *	*	1.189 *	*
Mammary tumor	1.006 *	0.254*	1.049 *	1.034 *
Breast cancer	−1.067 *	*	0.817 *	*
Morphology of testis	ns	ns	*	ns
Atrophy of testis	ns	ns	0.711*	ns
*Neurological Diseases and Disorders (Brain)*				
Brain tumor	0.644 *	0.152 *	*	0.378 *
Congenital neurological disorder	−2.456 *	−1.538 *	*	3.619 *
Nervous system neoplasm	0.689 *	0.566 *	−0.283 *	0.304 *
Central nervous system cancer	0.875 *	0.069 *	*	2 *
Central nervous system solid tumor	0.229 *	0.497 *	*	0.218 *
Locomotion	ns	−0.954 *	ns	0.398 *
Learning	ns	2.878 *	ns	−3.582 *
Proliferation of neuronal cells	ns	1.739 *	ns	−2.969 *
Cognition	ns	2.939 *	ns	−3.828 *
Quantity of neurons	ns	0.443 *	ns	−1.984 *
Development of neurons	ns	1.693 *	ns	−3.528 *
Development central nervous system	ns	1.815 *	ns	−3.282 *
Memory	ns	1.544 *	ns	−1.571 *
*Endocrine System Diseases and Disorders (Gonad)*				
Thyroid carcinoma	*	*	*	*
Nonpituitary endocrine tumor	0.404 *	*	*	*
Endocrine gland tumor	0.017 *	*	−0.401 *	ns
Endocrine carcinoma	*	*	*	*

## Data Availability

Data are contained within the article and Supplementary Materials.

## Supplementary Materials

The supporting information can be downloaded at: https://www.mdpi.com/article/10.3390/ijms242216212/s1.

## References

[1] Du L., Batterman S., Godwin C., Rowe Z., Chin J.-Y. (2015). Air Exchange Rates and Migration of VOCs in Basements and Residences. Indoor Air.

[2] McDonald B.C., de Gouw J.A., Gilman J.B., Jathar S.H., Akherati A., Cappa C.D., Jimenez J.L., Lee-Taylor J., Hayes P.L., McKeen S.A. (2018). Volatile Chemical Products Emerging as Largest Petrochemical Source of Urban Organic Emissions. Science.

[3] Porada E., Szyszkowicz M. (2016). UNMIX Methods Applied to Characterize Sources of Volatile Organic Compounds in Toronto, Ontario. Toxics.

[4] Hsu C.-Y., Chiang H.-C., Shie R.-H., Ku C.-H., Lin T.-Y., Chen M.-J., Chen N.-T., Chen Y.-C. (2018). Ambient VOCs in Residential Areas near a Large-Scale Petrochemical Complex: Spatiotemporal Variation, Source Apportionment and Health Risk. Environ. Pollut. Barking Essex 1987.

[5] Saravanakumar K., Sivasantosh S., Sathiyaseelan A., Sankaranarayanan A., Naveen K.V., Zhang X., Jamla M., Vijayasarathy S., Vishnu Priya V., MubarakAli D. (2022). Impact of Benzo[a]Pyrene with Other Pollutants Induce the Molecular Alternation in the Biological System: Existence, Detection, and Remediation Methods. Environ. Pollut..

[6] Benzene|Toxzine|ATSDR. https://www.atsdr.cdc.gov/sites/toxzine/benzene_toxzine.html.

[7] Duarte-Davidson R. (2001). Benzene in the Environment: An Assessment of the Potential Risks to the Health of the Population. Occup. Environ. Med..

[8] Rowe B.L., Toccalino P.L., Moran M.J., Zogorski J.S., Price C.V. (2007). Occurrence and Potential Human-Health Relevance of Volatile Organic Compounds in Drinking Water from Domestic Wells in the United States. Environ. Health Perspect..

[9] Zhong L., Batterman S., Milando C.W. (2019). VOC Sources and Exposures in Nail Salons: A Pilot Study in Michigan, USA. Int. Arch. Occup. Environ. Health.

[10] Lamplugh A., Harries M., Xiang F., Trinh J., Hecobian A., Montoya L.D. (2019). Occupational Exposure to Volatile Organic Compounds and Health Risks in Colorado Nail Salons. Environ. Pollut. Barking Essex 1987.

[11] Chong N.S., Abdulramoni S., Patterson D., Brown H. (2019). Releases of Fire-Derived Contaminants from Polymer Pipes Made of Polyvinyl Chloride. Toxics.

[12] U.S. Food&Drug Administration (2022). FDA Alerts Drug Manufacturers to the Risk of Benzene Contamination in Certain Drugs.

[13] Pal V.K., Lee S., Naidu M., Lee C., Kannan K. (2022). Occurrence of and Dermal Exposure to Benzene, Toluene and Styrene Found in Hand Sanitizers from the United States. Environ. Int..

[14] Pal V.K., Lee S., Kannan K. (2023). Occurrence of and Dermal Exposure to Benzene, Toluene and Styrene in Sunscreen Products Marketed in the United States. Sci. Total Environ..

[15] Nakai J.S., Chu I., Li-Muller A., Aucoin R. (1997). Effect of Environmental Conditions on the Penetration of Benzene through Human Skin. J. Toxicol. Environ. Health.

[16] U.S. Environmental Protection Agency Benzene CASRN 71-43-2|DTXSID3039242|IRIS|US EPA, ORD. https://cfpub.epa.gov/ncea/iris2/chemicallanding.cfm?substance_nmbr=276#:~:text=Benzene%20is%20classified%20as%20a,Risk%20Assessment%20Guidelines%20of%201986.

[17] Wen Q., Boshier P., Myridakis A., Belluomo I., Hanna G.B. (2020). Urinary Volatile Organic Compound Analysis for the Diagnosis of Cancer: A Systematic Literature Review and Quality Assessment. Metabolites.

[18] Hanna G.B., Boshier P.R., Markar S.R., Romano A. (2019). Accuracy and Methodologic Challenges of Volatile Organic Compound-Based Exhaled Breath Tests for Cancer Diagnosis: A Systematic Review and Meta-Analysis. JAMA Oncol..

[19] Rana I., Dahlberg S., Steinmaus C., Zhang L. (2021). Benzene Exposure and Non-Hodgkin Lymphoma: A Systematic Review and Meta-Analysis of Human Studies. Lancet Planet. Health.

[20] Odutola M.K., Benke G., Fritschi L., Giles G.G., van Leeuwen M.T., Vajdic C.M. (2021). A Systematic Review and Meta-Analysis of Occupational Exposures and Risk of Follicular Lymphoma. Environ. Res..

[21] D’Andrea M.A., Reddy G.K. (2018). Health Risks Associated With Benzene Exposure in Children: A Systematic Review. Glob. Pediatr. Health.

[22] Smargiassi A., Goldberg M.S., Wheeler A.J., Plante C., Valois M.-F., Mallach G., Kauri L.M., Shutt R., Bartlett S., Raphoz M. (2014). Associations between Personal Exposure to Air Pollutants and Lung Function Tests and Cardiovascular Indices among Children with Asthma Living near an Industrial Complex and Petroleum Refineries. Environ. Res..

[23] Choi Y.-H., Kim J.H., Lee B.-E., Hong Y.-C. (2014). Urinary Benzene Metabolite and Insulin Resistance in Elderly Adults. Sci. Total Environ..

[24] Cordiano R., Papa V., Cicero N., Spatari G., Allegra A., Gangemi S. (2022). Effects of Benzene: Hematological and Hypersensitivity Manifestations in Resident Living in Oil Refinery Areas. Toxics.

[25] Slama R., Thiebaugeorges O., Goua V., Aussel L., Sacco P., Bohet A., Forhan A., Ducot B., Annesi-Maesano I., Heinrich J. (2009). Maternal Personal Exposure to Airborne Benzene and Intrauterine Growth. Environ. Health Perspect..

[26] Cassidy-Bushrow A.E., Burmeister C., Lamerato L., Lemke L.D., Mathieu M., O’Leary B.F., Sperone F.G., Straughen J.K., Reiners J.J. (2020). Prenatal Airshed Pollutants and Preterm Birth in an Observational Birth Cohort Study in Detroit, Michigan, USA. Environ. Res..

[27] Kumar P., Singh A.B., Arora T., Singh S., Singh R. (2023). Critical Review on Emerging Health Effects Associated with the Indoor Air Quality and Its Sustainable Management. Sci. Total Environ..

[28] Klepeis N.E., Nelson W.C., Ott W.R., Robinson J.P., Tsang A.M., Switzer P., Behar J.V., Hern S.C., Engelmann W.H. (2001). The National Human Activity Pattern Survey (NHAPS): A Resource for Assessing Exposure to Environmental Pollutants. J. Expo. Anal. Environ. Epidemiol..

[29] Chatterjee N., Kim C., Im J., Kim S., Choi J. (2023). Mixture and Individual Effects of Benzene, Toluene, and Formaldehyde in Zebrafish (Danio Rerio) Development: Metabolomics, Epigenetics, and Behavioral Approaches. Environ. Toxicol. Pharmacol..

[30] Marchini S., Tosato M.L., Norberg-King T.J., Hammermeister D.E., Hoglund M.D. (1992). Lethal and Sublethal Toxicity of Benzene Derivatives to the Fathead Minnow, Using a Short-Term Test. Environ. Toxicol. Chem..

[31] Philibert D.A., Philibert C.P., Lewis C., Tierney K.B. (2016). Comparison of Diluted Bitumen (Dilbit) and Conventional Crude Oil Toxicity to Developing Zebrafish. Environ. Sci. Technol..

[32] Bérubé R., Gauthier C., Bourdin T., Bouffard M., Triffault-Bouchet G., Langlois V.S., Couture P. (2021). Lethal and Sublethal Effects of Diluted Bitumen and Conventional Oil on Fathead Minnow (*Pimephales promelas*) Larvae Exposed during Their Early Development. Aquat. Toxicol..

[33] Chatterjee S., Behnam Azad B., Nimmagadda S. (2014). The Intricate Role of CXCR4 in Cancer. Adv. Cancer Res..

[34] He J., Zang S., Liu N., Ji M., Ma D., Ji C. (2020). Epimedium Polysaccharides Attenuates Hematotoxicity by Reducing Oxidative Stress and Enhancing Immune Function in Mice Model of Benzene-Induced Bone Marrow Failure. Biomed. Pharmacother..

[35] Qiao Y., Hu H., Zhao Y., Jin M., Yang D., Yin J., Wu P., Liu W., Li J. (2023). Benzene Induces Spleen Injury through the B Cell Receptor Signaling Pathway. Ecotoxicol. Environ. Saf..

[36] Zhao J., Sui P., Wu B., Chen A., Lu Y., Hou F., Cheng X., Cui S., Song J., Huang G. (2021). Benzene Induces Rapid Leukemic Transformation after Prolonged Hematotoxicity in a Murine Model. Leukemia.

[37] Badham H.J., Winn L.M. (2010). In Utero Exposure to Benzene Disrupts Fetal Hematopoietic Progenitor Cell Growth via Reactive Oxygen Species. Toxicol. Sci..

[38] Varona A., Echevarria E., Irazusta J., Serrano R., Gil J., Casis L. (1998). Effects of Acute Benzene Exposure on Brain Enkephalin Immunostaining and Degradation. Neurotoxicol. Teratol..

[39] Koshko L., Scofield S., Debarba L., Stilgenbauer L., Sacla M., Fakhoury P., Jayarathne H., Perez-Mojica J.E., Griggs E., Lempradl A. (2023). Prenatal Benzene Exposure Alters Offspring Hypothalamic Development Predisposing to Metabolic Disease in Later Life. bioRxiv.

[40] Sun R., Liu M., Xu K., Pu Y., Huang J., Liu J., Zhang J., Yin L., Pu Y. (2022). Ferroptosis Is Involved in the Benzene-Induced Hematotoxicity in Mice via Iron Metabolism, Oxidative Stress and NRF2 Signaling Pathway. Chem. Biol. Interact..

[41] Badham H.J., Renaud S.J., Wan J., Winn L.M. (2010). Benzene-Initiated Oxidative Stress: Effects on Embryonic Signaling Pathways. Chem. Biol. Interact..

[42] Weisel C.P. (2010). Benzene Exposure: An Overview of Monitoring Methods and Their Findings. Chem. Biol. Interact..

[43] Howe K., Clark M.D., Torroja C.F., Torrance J., Berthelot C., Muffato M., Collins J.E., Humphray S., McLaren K., Matthews L. (2013). The Zebrafish Reference Genome Sequence and Its Relationship to the Human Genome. Nature.

[44] Wu C.-C., Blount J.R., Haimbaugh A., Heldman S., Shields J.N., Baker T.R. (2022). Evaluating Phenotypic and Transcriptomic Responses Induced by Low-Level VOCs in Zebrafish: Benzene as an Example. Toxics.

[45] Lex A., Gehlenborg N., Strobelt H., Vuillemot R., Pfister H. (2014). UpSet: Visualization of Intersecting Sets. IEEE Trans. Vis. Comput. Graph..

[46] Conway J.R., Lex A., Gehlenborg N. (2017). UpSetR: An R Package for the Visualization of Intersecting Sets and Their Properties. Bioinformatics.

[47] Baron J.A., Nichols H.B., Anderson C., Safe S. (2021). Cigarette Smoking and Estrogen-Related Cancer. Cancer Epidemiol. Biomark. Prev. Publ. Am. Assoc. Cancer Res. Cosponsored Am. Soc. Prev. Oncol..

[48] Wolff M.S., Collman G.W., Barrett J.C., Huff J. (1996). Breast Cancer and Environmental Risk Factors: Epidemiological and Experimental Findings. Annu. Rev. Pharmacol. Toxicol..

[49] Costantini A.S., Gorini G., Consonni D., Miligi L., Giovannetti L., Quinn M. (2009). Exposure to Benzene and Risk of Breast Cancer among Shoe Factory Workers in Italy. Tumori.

[50] Fenga C., Gangemi S., Costa C. (2016). Benzene Exposure Is Associated with Epigenetic Changes (Review). Mol. Med. Rep..

[51] Maronpot R.R. (1987). Ovarian Toxicity and Carcinogenicity in Eight Recent National Toxicology Program Studies. Environ. Health Perspect..

[52] Bahadar H., Mostafalou S., Abdollahi M. (2014). Current Understandings and Perspectives on Non-Cancer Health Effects of Benzene: A Global Concern. Toxicol. Appl. Pharmacol..

[53] Danysh H.E., Mitchell L.E., Zhang K., Scheurer M.E., Lupo P.J. (2015). Traffic-Related Air Pollution and the Incidence of Childhood Central Nervous System Tumors: Texas, 2001–2009. Pediatr. Blood Cancer.

[54] Mazzei A., Konstantinoudis G., Kreis C., Diezi M., Ammann R.A., Zwahlen M., Kühni C., Spycher B.D. (2022). Childhood Cancer and Residential Proximity to Petrol Stations: A Nationwide Registry-Based Case-Control Study in Switzerland and an Updated Meta-Analysis. Int. Arch. Occup. Environ. Health.

[55] Geist C.R., Drew K.L., Schoenheit C.M., Praed J.E. (1983). Learning Impairments Following Postnatal Exposure to Benzene. Percept. Mot. Skills.

[56] Lo Pumo R., Bellia M., Nicosia A., Micale V., Drago F. (2006). Long-Lasting Neurotoxicity of Prenatal Benzene Acute Exposure in Rats. Toxicology.

[57] Gonzalez-Casanova I., Stein A.D., Barraza-Villarreal A., Feregrino R.G., DiGirolamo A., Hernandez-Cadena L., Rivera J.A., Romieu I., Ramakrishnan U. (2018). Prenatal Exposure to Environmental Pollutants and Child Development Trajectories through 7 Years. Int. J. Hyg. Environ. Health.

[58] Fu Y., Hsiao J.-H.T., Paxinos G., Halliday G.M., Kim W.S. (2015). ABCA5 Regulates Amyloid-β Peptide Production and Is Associated with Alzheimer’s Disease Neuropathology. J. Alzheimer’s Dis. JAD.

[59] Bu P., Xiao Y., Hu S., Jiang X., Tan C., Qiu M., Huang W., Li M., Li Q., Qin C. (2022). Identification of ABCA5 among ATP-Binding Cassette Transporter Family as a New Biomarker for Colorectal Cancer. J. Oncol..

[60] Talibov M., Sormunen J., Hansen J., Kjaerheim K., Martinsen J.-I., Sparen P., Tryggvadottir L., Weiderpass E., Pukkala E. (2018). Benzene Exposure at Workplace and Risk of Colorectal Cancer in Four Nordic Countries. Cancer Epidemiol..

[61] Papuc S.M., Abela L., Steindl K., Begemann A., Simmons T.L., Schmitt B., Zweier M., Oneda B., Socher E., Crowther L.M. (2019). The Role of Recessive Inheritance in Early-Onset Epileptic Encephalopathies: A Combined Whole-Exome Sequencing and Copy Number Study. Eur. J. Hum. Genet. EJHG.

[62] Reuter M.S., Tawamie H., Buchert R., Hosny Gebril O., Froukh T., Thiel C., Uebe S., Ekici A.B., Krumbiegel M., Zweier C. (2017). Diagnostic Yield and Novel Candidate Genes by Exome Sequencing in 152 Consanguineous Families With Neurodevelopmental Disorders. JAMA Psychiatry.

[63] Yang S.H., Shrivastav A., Kosinski C., Sharma R.K., Chen M.-H., Berthiaume L.G., Peters L.L., Chuang P.-T., Young S.G., Bergo M.O. (2005). N-Myristoyltransferase 1 Is Essential in Early Mouse Development. J. Biol. Chem..

[64] Quintero-Rivera F., Leach N.T., de la Chapelle A., Gusella J.F., Morton C.C., Harris D.J. (2007). Is the Disruption of an N-Myristoyltransferase (NMT2) Associated with Hypoplastic Testes?. Am. J. Med. Genet. Part A.

[65] Williams P.A., Krug M.S., McMillan E.A., Peake J.D., Davis T.L., Cocklin S., Strochlic T.I. (2016). Phosphorylation of the RNA-Binding Protein Dazl by MAPKAP Kinase 2 Regulates Spermatogenesis. Mol. Biol. Cell.

[66] Huo M., Han H., Sun Z., Lu Z., Yao X., Wang S., Wang J. (2016). Role of IL-17 Pathways in Immune Privilege: A RNA Deep Sequencing Analysis of the Mice Testis Exposure to Fluoride. Sci. Rep..

[67] Farrugia A.J., Rodríguez J., Orgaz J.L., Lucas M., Sanz-Moreno V., Calvo F. (2020). CDC42EP5/BORG3 Modulates SEPT9 to Promote Actomyosin Function, Migration, and Invasion. J. Cell Biol..

[68] Furusato B., Mohamed A., Uhlén M., Rhim J.S. (2010). CXCR4 and Cancer. Pathol. Int..

[69] Yun J.W., Lee S., Ryu D., Park S., Park W.-Y., Joung J.-G., Jeong J. (2019). Biomarkers Associated with Tumor Heterogeneity in Prostate Cancer. Transl. Oncol..

[70] Diotel N., Vaillant C., Gueguen M.-M., Mironov S., Anglade I., Servili A., Pellegrini E., Kah O. (2010). Cxcr4 and Cxcl12 Expression in Radial Glial Cells of the Brain of Adult Zebrafish. J. Comp. Neurol..

[71] Chong S.W., Emelyanov A., Gong Z., Korzh V. (2001). Expression Pattern of Two Zebrafish Genes, Cxcr4a and Cxcr4b. Mech. Dev..

[72] Lim Y.S., Tang B.L. (2013). The Evi5 Family in Cellular Physiology and Pathology. FEBS Lett..

[73] Hoppenbrouwers I.A., Aulchenko Y.S., Ebers G.C., Ramagopalan S.V., Oostra B.A., van Duijn C.M., Hintzen R.Q. (2008). EVI5 Is a Risk Gene for Multiple Sclerosis. Genes Immun..

[74] Jacob B., Osato M., Yamashita N., Wang C.Q., Taniuchi I., Littman D.R., Asou N., Ito Y. (2010). Stem Cell Exhaustion Due to Runx1 Deficiency Is Prevented by Evi5 Activation in Leukemogenesis. Blood.

[75] Tang J., Ou J., Xu C., Yi C., Xue F., Xu L., Lai F., Tang J., Li S., Kang T. (2017). EVI5 Is a Novel Independent Prognostic Predictor in Hepatocellular Carcinoma after Radical Hepatectomy. Oncol. Rep..

[76] Blaker-Lee A., Gupta S., McCammon J.M., De Rienzo G., Sive H. (2012). Zebrafish Homologs of Genes within 16p11.2, a Genomic Region Associated with Brain Disorders, Are Active during Brain Development, and Include Two Deletion Dosage Sensor Genes. Dis. Model. Mech..

[77] Bertrand R.E., Wang J., Xiong K.H., Thangavel C., Qian X., Ba-Abbad R., Liang Q., Simões R.T., Sampaio S.A.M., Carss K.J. (2021). Ceramide Synthase TLCD3B Is a Novel Gene Associated with Human Recessive Retinal Dystrophy. Genet. Med. Off. J. Am. Coll. Med. Genet..

[78] Gistelinck C., Kwon R.Y., Malfait F., Symoens S., Harris M.P., Henke K., Hawkins M.B., Fisher S., Sips P., Guillemyn B. (2018). Zebrafish Type I Collagen Mutants Faithfully Recapitulate Human Type I Collagenopathies. Proc. Natl. Acad. Sci. USA.

[79] Cole W.G., Dalgleish R. (1995). Perinatal Lethal Osteogenesis Imperfecta. J. Med. Genet..

[80] Oestreich A.K., DeCata J.A., Akers J.D., Phillips C.L., Schulz L.C. (2020). Fecundity Is Impaired in a Mouse Model of Osteogenesis Imperfecta. Mol. Reprod. Dev..

[81] Underhill L.A., Barbarita C., Collis S., Tucker R., Lechner B.E. (2022). Association of Maternal Versus Fetal Ehlers-Danlos Syndrome Status with Poor Pregnancy Outcomes. Reprod. Sci..

[82] Lind J., Wallenburg H.C.S. (2002). Pregnancy and the Ehlers-Danlos Syndrome: A Retrospective Study in a Dutch Population. Acta Obstet. Gynecol. Scand..

[83] Sorokin Y., Johnson M.P., Rogowski N., Richardson D.A., Evans M.I. (1994). Obstetric and Gynecologic Dysfunction in the Ehlers-Danlos Syndrome. J. Reprod. Med..

[84] Castori M., Morlino S., Dordoni C., Celletti C., Camerota F., Ritelli M., Morrone A., Venturini M., Grammatico P., Colombi M. (2012). Gynecologic and Obstetric Implications of the Joint Hypermobility Syndrome (a.k.a. Ehlers–Danlos Syndrome Hypermobility Type) in 82 Italian Patients. Am. J. Med. Genet. Part A.

[85] Gan Q., Liu Q., Hu X., You C. (2017). Collagen Type I Alpha 2 (COL1A2) Polymorphism Contributes to Intracranial Aneurysm Susceptibility: A Meta-Analysis. Med. Sci. Monit. Int. Med. J. Exp. Clin. Res..

[86] Seger R., Krebs E.G. (1995). The MAPK Signaling Cascade. FASEB J. Off. Publ. Fed. Am. Soc. Exp. Biol..

[87] Paton E.L., Turner J.A., Schlaepfer I.R. (2020). Overcoming Resistance to Therapies Targeting the MAPK Pathway in BRAF-Mutated Tumours. J. Oncol..

[88] Koedoot E., Fokkelman M., Rogkoti V.-M., Smid M., van de Sandt I., de Bont H., Pont C., Klip J.E., Wink S., Timmermans M.A. (2019). Uncovering the Signaling Landscape Controlling Breast Cancer Cell Migration Identifies Novel Metastasis Driver Genes. Nat. Commun..

[89] Koshko L., Debarba L.K., Sacla M., de Lima J.B.M., Didyuk O., Fakhoury P., Sadagurski M. (2021). In Utero Maternal Benzene Exposure Predisposes to the Metabolic Imbalance in the Offspring. Toxicol. Sci. Off. J. Soc. Toxicol..

[90] Debarba L.K., Mulka A., Lima J.B.M., Didyuk O., Fakhoury P., Koshko L., Awada A.A., Zhang K., Klueh U., Sadagurski M. (2020). Acarbose Protects from Central and Peripheral Metabolic Imbalance Induced by Benzene Exposure. Brain. Behav. Immun..

[91] Poli D., Mozzoni P., Pinelli S., Cavallo D., Papaleo B., Caporossi L. (2022). Sex Difference and Benzene Exposure: Does It Matter?. Int. J. Environ. Res. Public. Health.

[92] Goizet C., Depienne C., Benard G., Boukhris A., Mundwiller E., Solé G., Coupry I., Pilliod J., Martin-Négrier M.-L., Fedirko E. (2011). REEP1 Mutations in SPG31: Frequency, Mutational Spectrum, and Potential Association with Mitochondrial Morpho-Functional Dysfunction. Hum. Mutat..

[93] Kolachana P., Subrahmanyam V.V., Meyer K.B., Zhang L., Smith M.T. (1993). Benzene and Its Phenolic Metabolites Produce Oxidative DNA Damage in HL60 Cells in Vitro and in the Bone Marrow in Vivo. Cancer Res..

[94] Murugesan K., Baumann S., Wissenbach D.K., Kliemt S., Kalkhof S., Otto W., Mögel I., Kohajda T., von Bergen M., Tomm J.M. (2013). Subtoxic and Toxic Concentrations of Benzene and Toluene Induce Nrf2-Mediated Antioxidative Stress Response and Affect the Central Carbon Metabolism in Lung Epithelial Cells A549. Proteomics.

[95] Nair R.R., Kerätär J.M., Autio K.J., Masud A.J., Finnilä M.A.J., Autio-Harmainen H.I., Miinalainen I.J., Nieminen P.A., Hiltunen J.K., Kastaniotis A.J. (2017). Genetic Modifications of Mecr Reveal a Role for Mitochondrial 2-Enoyl-CoA/ACP Reductase in Placental Development in Mice. Hum. Mol. Genet..

[96] Chen Z., Leskinen H., Liimatta E., Sormunen R.T., Miinalainen I.J., Hassinen I.E., Hiltunen J.K. (2009). Myocardial Overexpression of Mecr, a Gene of Mitochondrial FAS II Leads to Cardiac Dysfunction in Mouse. PLoS ONE.

[97] Hu R., Xu Y., Han B., Chen Y., Li W., Guan G., Hu P., Zhou Y., Xu Q., Chen L. (2022). MiR-202-3p Determines Embryo Viability during Mid-Blastula Transition. Front. Cell Dev. Biol..

