# Supplementary material for: Adult-Onset Transcriptomic Effects of Developmental Exposure to Benzene in Zebrafish (Danio rerio): Evaluating a Volatile Organic Compound of Concern
Source: Int J Mol Sci. 2023 Nov 11;24(22):16212. doi: 10.3390/ijms242216212 (PMC10671089; doi:10.3390/ijms242216212)
Supplement: Supplementary file 1 [file ijms-24-16212-s001.zip › Supplemental Tables final.pdf]

Supplemental Table S1. Differentially expressed genes and associated log2fold change values of benzene exposed zebrafish with p-values<0.05 from larval gene expression results (Wu et al., 2022) and novel adult gene expression results

	**0.1 ppm**					**1.0 ppm**				
**Gene**	Larval (Wu et al., 2022)	Adult Female Brain	Adult Female Gonad	Adult Male Brain	Adult Male Gonad	Larval (Wu et al., 2022)	Adult Female Brain	Adult Female Gonad	Adult Male Brain	Adult Male Gonad
reep1	1.82	1.08				1.7	1.06		-1.48	
mecr	1.97	-1.03				2.14				
nfkbiaa		0.90	1.09			1.70			1.01	
taf4a	0.52		1.04			0.5			-0.68	
grik1a						0.56		-0.76		
aspm			1.24			1.69		0.91		
mgp						0.53				0.95

Supplemental Table S2. The number of molecules in associated pathways based on benzene concentration, sex (F/M), and tissue type, brain (B) or Gonad (G).

	0.1 ppm				1.0 ppm			
	FB	FG	MB	MG	FB	FG	MB	MG
**Canonical Pathways**								
Insulin Secretion Pathway	48	14		20	29	5	38	6
Cardiac Hypertrophy Signaling (Enhanced)	64	26	1	11	39	5	51	11
S100 Family Signaling Pathway	21	9	4	39	36	3	68	12
**Diseases and Disorders**								
Cancer	2,162	520	98	1082	884	202	1329	258
Organismal injury and abnormalities	2,180	522	101	1100	894	204	1340	261
Endocrine system disorders	1,878	480	89	953	795	184	1,208	219
Gastrointestinal disease	1,938	491		956	827	189	1,228	
Neurological disease	1,607	402	85	782				191
Reproductive system disease			80			151	1,022	199
**Molecular and cellular function**								
Cellular assembly and organization	560	169			233	50	340	
Cellular function and maintenance	758				347	38	516	
Cell death and survival	823	216	439					
Cellular Development	678				344		463	
Protein synthesis		9	145					
**Physiological system development**								
Organismal survival	615	170				65	414	79
Nervous system development and function	562				312		392	
Tissue development	535				285		418	
Embryonic development	594		39	140		39		
Organismal development	816	200		306	357	45	589	105
Cardiovascular system development		117	17	152				55
Connective tissue development and function				167				

Supplemental Table S3. Sub-pathways associated with reproductive system, neurological, and endocrine system diseases and disorders altered in gonad or brain tissue after developmental exposure to 0.1 ppm or 1.0 ppm benzene. Activation z-score indicated numerically and p-value < 0.05 indicated by * with nf denoting 'not found' for the given condition.

Associated Pathway	0.1 ppm Female	1.0 ppm Female	0.1 ppm Male	1.0 ppm Male
Reproductive Diseases and Disorders (gonad)				
Malignant genitourinary solid tumor	nf	*	1.431*	2.219*
Genitourinary carcinoma	nf	*	1.233*	*
Death of embryo	-4.10*	nf	nf	nf
Genital tumor	-0.439*	1.154*	1.079*	2.59*
Pelvic tumor	-0.595*	1.154*	0.819*	2.581*
Genitourinary tumor	nf	0.683*	nf	2.353*
Malignant genitourinary solid tumor	nf	*	1.431*	2.219*
Anogenital cancer	-1.504*	nf	1.12*	nf
Breast or gastric cancer	-1.195*	*	1.077*	*
Breast or pancreatic cancer	-0.558*	*	1.161*	*
Uterine tumor	*	*	*	*
Endometrial cancer	*	*	*	*
Pelvic carcinoma	-1.982*	*	0.963*	*
Uterine cancer	*	*	*	*
Breast or colorectal cancer	-0.478*	*	0.506*	0.64*
Breast or ovarian carcinoma	-1.067*	*	1.066*	*
Breast or gynecological cancer	-1.387*	*	1.189*	*
Female genital tract cancer	*	*	*	*
Mammary tumor	1.006*	0.254*	1.049*	1.034*
Genital tract cancer	-1.076*	*	1.019*	*
Breast carcinoma	*	*	1.066*	*
Genital carcinoma	*	*	1.114*	*
Uterine carcinoma	*	*	*	*
Female genital carcinoma	*	*	*	*
Development of genital tumor	*	*	1.457*	*
Female genital neoplasm	0.651*	*	1.159*	*
Pelvic tumor	-0.595*	1.154*	0.819*	2.581*
Breast cancer	-1.067*	*	0.817*	*
Breast or ovarian cancer	-1.387*	*	0.835*	*
Invasive breast cancer	*	*	nf	*
Morphology of genital organ	*	nf	*	nf
Tumorigenesis of reproductive tract	0.651*	nf	1.159*	nf
Morphology of gonad	*	nf	nf	nf
Morphology of testis	nf	nf	*	nf
Atrophy of testis	nf	nf	0.711*	nf
Neurological Diseases and Disorders (brain)				
Brain tumor	0.644*	0.152*	*	0.378*
Congenital neurological disorder	-2.456*	-1.538*	*	3.619*

Gliomatosis cerebri	*	*	*	*
Glioma cancer	-0.533*	*	*	*
Brain oligodendroglioma	*	*	*	*
Nervous system neoplasm	0.689*	0.566*	-0.283*	0.304*
Grade 3-4 glioma cancer	*	*	*	na*
Familial central nervous system disease	0.784*	*	*	*
Familial neurological disorder	0.686*	*	*	1.39*
Grade 4 high grade glioma	*	*	*	*
Brain cancer	1.063*	0.294*	*	1.732*
Brain glioma	*	*	*	*
Familial encephalopathy	0.784*	*	*	*
Brain astrocytoma	*	*	*	*
Autosomal recessive neurological disorder	*	*	*	*
High grade astrocytoma	-0.607*	*	*	*
Central nervous system cancer	0.875*	0.069*	*	2*
Central nervous system solid tumor	0.229*	0.497*	*	0.218*
Grade 3 malignant glioma	*	*	*	*
Early-onset encephalopathy	-1.583*	-1.262*	*	3.745*
Grade 4 astrocytoma	*	*	*	*
Locomotion	nf	-0.954*	nf	0.398*
Learning	nf	2.878*	nf	-3.582*
Proliferation of neuronal cells	nf	1.739*	nf	-2.969*
Cognition	nf	2.939*	nf	-3.828*
Quantity of neurons	nf	0.443*	nf	-1.984*
Development of neurons	nf	1.693*	nf	-3.528*
Development of central nervous system	nf	1.815*	nf	-3.282*
Outgrowth of neurons	nf	1.335*	nf	-3.115*
Memory	nf	1.544*	nf	-1.571*
Endocrine System Diseases and Disorders (gonad)				
Thyroid carcinoma	*	*	*	*
Nonpituitary endocrine tumor	0.404*	*	*	*
Endocrine carcinoma	*	*	*	*
Endocrine gland tumor	0.017*	*	-0.401*	nf
Thyroid gland tumor	*	*	*	nf
Thyroid cancer	nf	nf	*	*
Activation of thyroid gland	nf	*	nf	nf
Development of endocrine region of pancreas	nf	nf	nf	*
Development of pancreas	nf	nf	nf	*
Development of endocrine gland	nf	nf	nf	*
Differentiated thyroid cancer	nf	nf	nf	*
Thyroid gland nonmedullary carcinoma	nf	nf	nf	*
Papillary thyroid carcinoma	nf	nf	nf	*
